# Reduction in accuracy of genomic prediction for ordered categorical data compared to continuous observations

**DOI:** 10.1186/1297-9686-46-37

**Published:** 2014-06-09

**Authors:** Kadir Kizilkaya, Rohan L Fernando, Dorian J Garrick

**Affiliations:** 1 Department of Animal Science, Iowa State University, Ames IA 50011, USA; 2 Department of Animal Science, Adnan Menderes University, Aydin 09100, Turkey; 3 Institute of Veterinary, Animal and Biomedical Sciences, Massey University, Palmerston North, New Zealand

## Abstract

**Background:**

Accuracy of genomic prediction depends on number of records in the training population, heritability, effective population size, genetic architecture, and relatedness of training and validation populations. Many traits have ordered categories including reproductive performance and susceptibility or resistance to disease. Categorical scores are often recorded because they are easier to obtain than continuous observations. Bayesian linear regression has been extended to the threshold model for genomic prediction. The objective of this study was to quantify reductions in accuracy for ordinal categorical traits relative to continuous traits.

**Methods:**

Efficiency of genomic prediction was evaluated for heritabilities of 0.10, 0.25 or 0.50. Phenotypes were simulated for 2250 purebred animals using 50 QTL selected from actual 50k SNP (single nucleotide polymorphism) genotypes giving a proportion of causal to total loci of.0001. A Bayes C *π* threshold model simultaneously fitted all 50k markers except those that represented QTL. Estimated SNP effects were utilized to predict genomic breeding values in purebred (n = 239) or multibreed (n = 924) validation populations. Correlations between true and predicted genomic merit in validation populations were used to assess predictive ability.

**Results:**

Accuracies of genomic estimated breeding values ranged from 0.12 to 0.66 for purebred and from 0.04 to 0.53 for multibreed validation populations based on Bayes C *π* linear model analysis of the simulated underlying variable. Accuracies for ordinal categorical scores analyzed by the Bayes C *π* threshold model were 20% to 50% lower and ranged from 0.04 to 0.55 for purebred and from 0.01 to 0.44 for multibreed validation populations. Analysis of ordinal categorical scores using a linear model resulted in further reductions in accuracy.

**Conclusions:**

Threshold traits result in markedly lower accuracy than a linear model on the underlying variable. To achieve an accuracy equal or greater than for continuous phenotypes with a training population of 1000 animals, a 2.25 fold increase in training population size was required for categorical scores fitted with the threshold model. The threshold model resulted in higher accuracies than the linear model and its advantage was greatest when training populations were smallest.

## Background

Recent innovation in high-throughput Single Nucleotide Polymorphism (SNP) genotyping technology has made SNP chips commercially available for most livestock species, including cattle, sheep, pigs, horses and chickens [1]. Bayesian linear regression models (Bayes A, B and C, Bayesian LASSO, machine learning methods) have made jointly fitting all SNP effects feasible for genomic prediction and genome-wide association analyses. Application of genome-wide Bayesian regression models, as described by Meuwissen et al. [2], require simultaneous estimation of marker effects across the entire genome using genotypes and phenotypes of animals in a training population, before prediction of breeding values of selection candidates or animals in a validation population based on their marker genotypes and estimated marker effects from the training data analyses. Findings from recent genome-wide studies investigating the effects of marker density, heritability, number of observations and relationships among the individuals in training population using simulated or actual data have indicated that genomic prediction is often superior to pedigree-based BLUP prediction in terms of accuracy of prediction [3-7].

For continuous traits, the factors that affect the accuracy of genomic prediction by Bayesian linear regression models have been studied using simulated [8-11] and field [6,12] data analyses in purebred (PB) and multibreed (MB) populations. Results from those studies have demonstrated that accuracy of genomic estimated breeding values (GEBV) depend on the number of records in the training population, the heritability of the trait, the effective population size, the size of the genome, the density of markers, the genetic architecture of the trait, and the extent of relatedness between training and validation populations [1,11,13]. Calus et al. [4] investigated the accuracy of GEBV produced by genomic selection in a simulation study using different map densities and haplotype structures in a 3 Morgan genome in an unselected outbred population, and found that the greatest benefit of genomic selection was for traits with a low heritability.

Categorical scores are often recorded because they are easier to obtain than continuous observations on the same trait. Many traits of low heritability have ordered categorical scores, such as susceptibility or resistance to a disease and reproductive traits like calving difficulty. In theory, methods that are used to analyze continuously distributed traits are not optimal for the analysis of ordinal categorical traits [14]. Wright [15] developed the threshold concept to map a normally distributed underlying variable to the observed ordered categorical phenotypes. In a threshold model, the phenotype is assumed to be the visible expression of an underlying continuous variable rendered discrete via a set of fixed thresholds [16]. Gianola and Foulley [17], and Harville and Mee [18] developed the threshold mixed effects model, which has become popular for pedigree-based genetic evaluation of ordinal categorical traits.

Due to the importance of ordinal categorical scores in animal production systems and the benefits of genomic selection, Kizilkaya et al. [19] used a BayesC threshold model to analyze the ordinal categorical trait of Infectious Bovine Keratoconjunctivitis in Angus beef cattle. The same model was used for the genome-wide association analysis of first service conception and pregnancy in Brangus heifers [20] and for insect bite hypersensitivity [21]. Furthermore, BayesA, Bayesian LASSO and two machine learning methods [22], as well as BayesB [23] have been extended to the threshold model in order to obtain genomic predictions of breeding values for binary traits.

It is expected that the realized accuracy for an ordinal categorical trait will be lower than that predicted from theory for a continuous trait in the same population with the same genetic architecture and heritability. The objective of this study was to use computer simulation to quantify that reduction in accuracy for an ordinal categorical trait relative to a continuous trait across the range of commonly encountered heritabilities in purebred and multibreed beef cattle populations.

## Methods

In order to quantify the reduction in accuracy of prediction of breeding values for an ordinal categorical trait relative to a continuous trait, underlying and ordinal categorical phenotypes for a training population, and true breeding values for training and validation populations were simulated using real SNP data from two beef cattle resource populations. The SNP genotypes were obtained using DNA samples extracted from semen or hair samples and did not require an approved animal use and care protocol. These simulations are described in greater detail below.

### Marker genotypes for training and validation populations

High-density genotypes 53 367 (50k) were obtained from the two resource populations using the Bovine SNP50 Infinium II BeadChip (Illumina, Inc., San Diego CA). The first resource population included 2250 purebred (PT) American Angus cattle [24] and was used as the training population.

The second population included a subsample of 924 animals from the multibreed Carcass Merit Project [25], and was used as the validation population (MV). In that population, Angus, Brahman, Charolais, Hereford, Limousin, Maine-Anjou, Shorthorn, South Devon AI sires were mated to commercial cows, and DNA samples and phenotypes were collected from 239 Angus-, 10 Brahman-, 183 Charolais-, 78 Hereford-, 45 Limousin-, 137 Maine-Anjou-, 97 Shorthorn-, and 135 South Devon-sired steer offspring [25]. The 239 purebred Angus animals in the MV population were used as a purebred Angus validation (PV) population in the project.

SNP covariate values of 0, 1, or 2, representing the number of B alleles in the Illumina A/B allele calls, were available for each locus. Missing genotypes represented less than 0.2% of total observations and were replaced with average covariate values for that locus. All genotypes were retained for analysis, regardless of minor allele frequency in the genotype data set [10].

### Simulation of underlying and categorical phenotypes for the training population

The observed 50k genotypes from the PT population were used to simulate *n* = 2250 underlying (latent) phenotypes with heritability of 10%, 25% or 50%. Then, *n* = 1000 samples were selected randomly from 2250 observations in order to create smaller training populations.

The underlying phenotypic value of each animal was simulated using the model:

(1) $$l_{i}=x_{i}^{\prime }\beta +\overset{K}{\underset{j=1}{\sum }}z_{\text{ij}}\alpha_{j}\delta_{j}+e_{i}\;i=1,\ldots ,n$$

where *l*_*i*_ is the underlying phenotypic value of animal *i*, ***β*** is a vector of fixed effects sampled from the standard normal distribution, **x***i*′ is the incidence row vector of animal *i*, relating its fixed effects to the underlying phenotypic value, *δ*_*j*_ is a Bernoulli random variable indicating the selection of locus *j* as a QTL from the observed SNP loci with fixed probability (1- *π*) where *π* = 0.999, *α*_*j*_ is the random substitution effect for locus *j*, sampled from a normal distribution with mean equal to 0 and variance $\sigma_{\alpha }^{2}=\frac{\sigma_{g}^{2}}{K(1-\pi )2\bar{\text{pq}}}$, *K* is the number of SNPs and $2\bar{\text{pq}}$ is the mean heterozygosity of the SNPs, *z*_*i* *j*_ is the covariate with 0, 1 or 2 at locus *j* for animal *i*, and *e*_*i*_ is the random residual effect, which has a standard normal distribution with mean equal to 0 and variance $\sigma_{e}^{2}$ = 1. The value of $\bar{\text{pq}}$ was estimated from all SNP loci in the PT population, and values of 0.12, 0.33 or 1 were assigned to $\sigma_{g}^{2}$ so that the expected heritabilities (*h*^2^) of underlying variables were 0.10, 0.25 or 0.50 [26].

Thirty-two replicates of the data were generated for each of three heritability scenarios. One fixed class effect with three levels was generated from the standard normal distribution in the simulation. Levels of fixed effects were randomly assigned to individuals in generating the underlying phenotypic values. The fixed effects (***β***), loci representing QTL (*δ*_*j*_=1) and substitution effects (*α*_*j*_) were sampled independently in each replicate.

In order to generate ordinal categorical phenotypes with four (*y*_4*i*_) categories, the underlying phenotypic values were mapped to ordinal categorical phenotypes based on the following threshold (*τ*) parameters:

(2) $$y_{4i}=\{\begin{array}{ll} 1 & \text{if}\;-\infty <l_{i}\leq \tau_{1},\\ 2 & \text{if}\;\tau_{1}<l_{i}\leq \tau_{2},\\ 3 & \text{if}\;\tau_{2}<l_{i}\leq \tau_{3},\\ 4 & \text{if}\;\;\tau_{3}<l_{i}\leq \mathrm{\infty .} \end{array}$$

**Figure 1:**
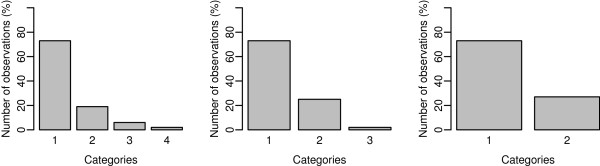
Distribution of observations by categories.

The threshold values were *τ*_1_=0.61, *τ*_2_=1.41, and *τ*_3_=2.05, which correspond to calving categories of no assistance, minor assistance, major assistance and caesarian section with frequencies of 73, 19, 6, and 2%, as observed in a Gelbvieh population [27] (Figure 1).

Two (*y*_2*i*_) and three (*y*_3*i*_)-category ordinal data sets were created by combining categories in the four-category ordinal data sets as follows:

(3) $$\;y_{2i}=\{\begin{array}{ll} 1 & \text{if}\;\;y_{4i}=1,\\ 2 & \text{if}\;\;2\leq y_{4i}\leq 4, \end{array}\;\text{and}\;y_{3i}=\{\begin{array}{ll} 1 & \text{if}\;\;y_{4i}=1,\\ 2 & \text{if}\;\;2\leq y_{4i}\leq 3,\\ 3 & \text{if}\;\;y_{4i}=4. \end{array}$$

### Models of analysis

#### Bayes C *π* threshold model

The threshold model [15] assumes that ordinal categorical data are determined by unobserved underlying continuous (**l**) variables and a set of unknown fixed thresholds, *τ*_1_<*τ*_2_<…<*τ*_*c*−1_, where *c* is the number of mutually exclusive, ordered categories. More specifically, the ordinal categorical score (*y*_*i*_) for animal *i* is assumed to be determined by the following:

(4) $$p(y_{i}=j|l_{i},\tau )=I(\tau_{j-1}<l_{i}<\tau_{j})I(y_{i}=j),$$

where *l*_*i*_ is the underlying variable of animal *i*, and *I*(.) is an indicator function taking the value 1 when expression (.) is true and 0 otherwise.

Marker-based binomial or ordinal threshold models were developed by modifying the Bayes C model described by Kizilkaya et al. [10], with the underlying variable for animal *i* modeled as follows:

(5) $$l_{i}=x_{i}^{\prime }\beta +\overset{K}{\underset{k=1}{\sum }}z_{\text{ik}}a_{k}+e_{i},$$

where ***β*** is a *p*x1 vector of fixed effects, **x***i*′ is a known incidence row vector corresponding to fixed effects in ***β***, *K* is the number of SNP loci in the genotype file, *z*_*i* *k*_ is the covariate (0, 1 or 2) at locus *k* for animal *i*, ***a***^′^= [*a*_1_,…,*a*_*k*_] is a *K*x1 vector of random substitution effects for *K* loci, and $e_{i}∼N(0,\sigma_{e}^{2})$ is a random residual. It was assumed that, given the location parameters ***β*** and ***a***, the underlying variable *l*_*i*_ of animal *i* is conditionally independent and distributed as

(6) $$l_{i}|\beta ,a∼N(x_{i}^{\prime }\beta +\overset{K}{\underset{k=1}{\sum }}z_{\text{ik}}a_{k},\sigma_{e}^{2}).$$

The joint posterior density of ***β***, ***a***, ***τ***, $\sigma_{a}^{2}$ and the underlying variable **l**[28] is given by:

(7) $$\begin{array}{lll} \;p\left(\beta ,a,l,\tau ,\sigma_{a}^{2}|y\right) & \propto p\left(y|\beta ,a,l,\tau ,\sigma_{a}^{2}\right)p\left(\beta ,a,l,\tau ,\sigma_{a}^{2}\right)\; & \;\\ & =p\left(y|l,\tau \right)p\left(l|\beta ,a\right)p\left(\beta ,a,\tau ,\sigma_{a}^{2}\right)\;\\ & =\overset{n}{\underset{i=1}{\prod }}\left[I\left(\tau_{j-1}<l_{i}<\tau_{j}\right)I\left(y_{i}=j\right)\right]\; & \;\\ & \;\times \left[\overset{n}{\underset{i=1}{\prod }}p\left(l|\beta ,a\right)\right]p\left(\beta ,a,\tau ,\sigma_{a}^{2}\right)\; & \; \end{array}$$

To ensure identifiability in the threshold model, $\sigma_{e}^{2}$ and the first threshold (*τ*_1_) were set to 1 and 0, respectively.

Flat prior distributions were assigned for the fixed effects ***β***. In Bayes C *π* threshold model, the prior for *a*_*k*_ follows a mixture distribution as:

(8) $$a_{k}|\pi ,\sigma_{a}^{2}=\{\begin{array}{ll} 0 & \text{with probability}\pi ,\\ ∼N(0,\sigma_{a}^{2}) & \text{with probability}(1-\pi ), \end{array}$$

where *π* is the probability that a SNP has no effect on the trait. The parameter *π* was treated as unknown with uniform *U*(0, 1) prior.

The variance $\sigma_{a}^{2}$ was a priori assumed to be a scaled inverse *Chi-Square* with degrees of freedom *ν* = 4 and scale parameter $S_{a}^{2}=\frac{\sigma_{g}^{2}(\nu -2)}{K(1-\pi )2\bar{\text{pq}}\nu }$. A flat prior was assumed for the thresholds.

The fixed effects *β*_*m*_ were sampled from

(9) $$\beta_{m}∼N({\hat{\beta }}_{m},{\left(x_{m}^{\prime }x_{m}\right)}^{-1})$$

where ${\hat{\beta }}_{m}=\frac{x_{m}^{\prime }(l-[X_{-m}\beta_{-m}+\overset{K}{\underset{k=1}{\sum }}Z_{k}a_{k}])}{x_{m}^{\prime }x_{m}}$ and **X**_−*m*_ is matrix **X** with the *m*th column deleted, and ***β***_−*m*_ is vector ***β*** with *m*th element deleted.

The full conditional posterior distribution for *a*_*k*_ was

(10) $$a_{k}|\pi ,\sigma_{a}^{2(t)}=\{\begin{array}{ll} 0 & \sigma_{a}^{2(t)}=0,\\ ∼N(\frac{{\text{z}}_{k}^{\prime }r_{k}}{C_{k}},C_{k}^{-1}) & \sigma_{a}^{2(t)}>0, \end{array}$$

where $r_{k}=l-[X\beta +\overset{K}{\underset{k^{\prime }\neq k}{\sum }}z_{k^{\prime }}a_{k^{\prime }}]$ and $C_{k}=z_{k}^{\prime }z_{k}+{\left(\sigma_{a}^{2}\right)}^{-1}$.

The common effect variance $\sigma_{a}^{2}$ was sampled from a scaled inverse *Chi-Square* with degrees of freedom $\tilde{\nu }=\nu +\nu_{M}^{\left(t\right)}$ and scale $\tilde{S_{a}^{2}}=\frac{\nu S_{a}^{2}+\overset{K}{\underset{k=1}{\sum }}a_{k}^{2}}{\tilde{\nu }}$, where $\nu_{M}^{\left(t\right)}$ is the number of SNPs fitted in iteration *t*.

The full conditional posterior distribution of the underlying variable was

(11) $$\;p(l_{i}|\beta ,a,\tau ,l_{-i},y)\propto p(l_{i}|\beta ,a)\left(I(\tau_{j-1}<l_{i}<\tau_{j})I(y_{i}=j)\right).$$

This density is a truncated *Normal* distribution with mean $\left({\text{x}}_{i}^{\prime }\beta +\overset{K}{\underset{k=1}{\sum }}z_{\text{ik}}a_{k}\right)$ and variance = 1. A Metropolis-Hastings scheme with the proposal distribution of truncated *normal*, which was presented by Cowles [29], was used to generate the samples for elements of ***τ*** in the ordinal setting.

The parameter of *π* was sampled from *Beta*(*K*−*M*^(*t*)^+1,*M*^(*t*)^+1), where *M*^(*t*)^ is the number of SNPs fitted in the model for iteration *t*. The starting value of *π* was 0.5.

#### Bayes C *π* linear model

Simulated liabilities **l** and binomial and ordinal categorical (*y*) data were modeled and analyzed as continuous traits using the linear model as follows,

(12) $$\tilde{y_{i}}=x_{i}^{\prime }\beta +\overset{K}{\underset{k=1}{\sum }}z_{\text{ik}}a_{k}+e_{i},$$

where $\tilde{y_{i}}$ is *l*_*i*_ or *y*_*i*_ for animal *i*, *e*_*i*_ was normally distributed residuals with mean = 0 and unknown variance $\sigma_{e}^{2}$[10].

### Markov chain Monte Carlo implementation

The 50k genotypes, excluding the 50 loci sampled as QTL (50k-QTL), were used to analyze each replicate data set with simulated underlying variables (*l*) and ordinal categorical phenotypes (*y*_2_,*y*_3_, and *y*_4_). SNP effects were estimated within PT (*n* = 1000 and 2250) training populations and used to predict genetic merit in the PV and MV validation populations. These procedures were implemented by Gibbs sampling in the GenSel software [30] applied to each replicated data set within each heritability scenario, by defining a burn-in period of 5000 Markov chain Monte Carlo (MCMC) cycles before saving samples from each of an additional 40 000 MCMC cycles.

### Genomic prediction and accuracy calculation

In training and validation populations, the true ($g_{i_{p}}$) and estimated (${ĝ}_{i_{p|\text{PT}}}$) genomic merits of animal *i* were calculated as

(13) $$g_{i_{p}}=\overset{Q}{\underset{q=1}{\sum }}z_{\text{iq}}\alpha_{q}\;\text{and}\;{ĝ}_{i_{p|\text{PT}}}=\overset{K}{\underset{k=1}{\sum }}z_{\text{ik}}{â}_{k}$$

where *p* is the PV or MV population, *z*_*i* *q*_ is the covariate (0, 1 or 2) at QTL locus *q* for animal *i* in population *p*, *z*_*i* *k*_ is the covariate (0, 1 or 2) at locus *k* for animal *i* in population *p*, *α*_*q*_ is the true value of the substitution effect at QTL locus *q*, and ${â}_{k}$ is the posterior mean of the substitution effect at locus *k*. In order to quantify the accuracy of prediction in validation populations, the sample covariance between the predicted (${\hat{g}}_{p|\text{PT}}$) and true (**g**_*p*_) additive genetic merits and their sample variances from breeds were pooled according to their respective degrees of freedom.

## Results and discussion

In this study, the realistic marker panel, 50k without QTL, was used for analyses of continuous and categorical phenotypes. The estimates of *π* varied depending on heritabilities, training population size, model of analysis and number of categories (Table 1). These results indicated a significant decreasing trend in estimates of *π* with increasing heritability and training population size. Habier et al. [31] investigated the estimation of *π* using a Bayes C *π* linear model in relation to number of training individuals, number of QTL and distribution of QTL effects. They observed decreasing trends for estimates of *π* with increasing training data size. Wolc et al. [7] studied the evaluation of accuracy of GEBV for economically important traits measured at early or late ages in a closed population of layer chickens over five successive generations using a Bayes C *π* linear model, and found that accuracy of GEBV increased with the size of the training data, moreso for traits with low estimates of *π* and high heritability. Also, a higher accuracy of GEBV for traits with high estimates of *π* was observed.

**Table 1:** **Estimates of *π * from Bayes C *π * analysis in the Angus training (PT) population**

**Type of phenotype**	**Model**	***h***^**2**^	***c***	**n = 1000**	**n = 2250**
Continuous	Linear	0.10	-	0.99982 ± 0.00002	0.99985 ± 0.00002
		0.25	-	0.99972 ± 0.00003	0.99940 ± 0.00006
		0.50	-	0.99903 ± 0.00007	0.99861 ± 0.00008
Categorical	Threshold	0.10	2	0.99700 ± 0.00159	0.99992 ± 0.00001
			3	0.99636 ± 0.00257	0.99992 ± 0.00001
			4	0.99881 ± 0.00053	0.99991 ± 0.00001
		0.25	2	0.99955 ± 0.00010	0.99976 ± 0.00004
			3	0.99939 ± 0.00019	0.99973 ± 0.00004
			4	0.99949 ± 0.00015	0.99973 ± 0.00004
		0.50	2	0.99562 ± 0.00112	0.99894 ± 0.00012
			3	0.99583 ± 0.00116	0.99900 ± 0.00008
			4	0.99319 ± 0.00258	0.99894 ± 0.00009
Categorical	Linear	0.10	2	0.99989 ± 0.00001	0.99995 ± 0.00001
			3	0.99987 ± 0.00001	0.99995 ± 0.00001
			4	0.99985 ± 0.00002	0.99994 ± 0.00001
		0.25	2	0.99992 ± 0.00001	0.99993 ± 0.00001
			3	0.99992 ± 0.00001	0.99992 ± 0.00001
			4	0.99991 ± 0.00001	0.99990 ± 0.00001
		0.50	2	0.99990 ± 0.00001	0.99981 ± 0.00002
			3	0.99988 ± 0.00001	0.99976 ± 0.00002
			4	0.99984 ± 0.00001	0.99967 ± 0.00003

The true value of *π* was 0.999.

**Figure 2:**
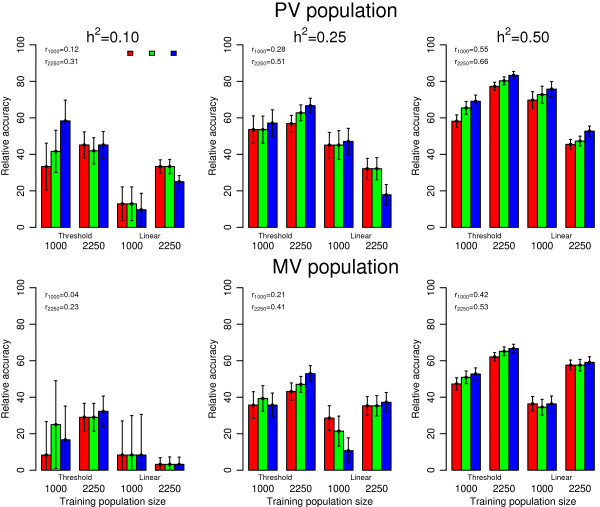
**Accuracies of GEBV for a categorical trait relative to a continuous trait.** Accuracies are given by validation population, heritability, training population size, model of analysis and the number of categories.

**Figure 3:**
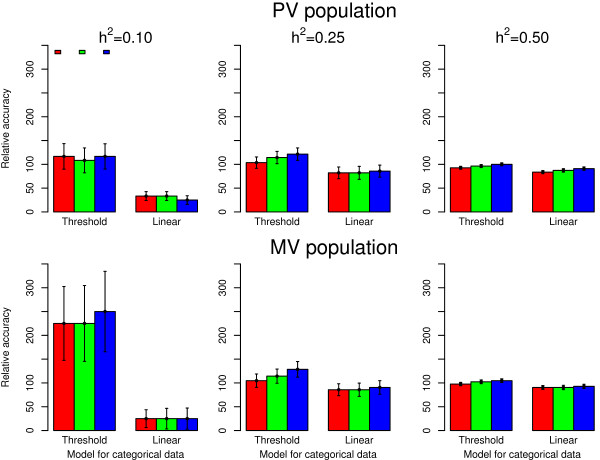
**Accuracies of GEBV for a categorical trait relative to a continuous trait in a smaller population.** The population size was 2250 for the categorical trait and was 1000 for the continuous trait.

Table 2 shows the accuracies of GEBV for continuous and ordinal categorical phenotypes across training population size, heritability, model of analysis and number of categories. For continuous phenotypes, accuracies ranged from 0.12 to 0.66 for PV and from 0.04 to 0.53 for MV validation populations. For ordinal categorical scores, accuracies ranged from 0.04 to 0.55 for PV and from 0.01 to 0.44 for MV based on the analysis of threshold model, and from 0.04 to 0.50 for PV and from 0.01 to 0.39 for MV based on the analysis using the linear model on categorical scores. The results in Table 2 indicate that genome-wide analysis of an ordinal categorical phenotype resulted in a substantially lower accuracy of GEBV than the analysis of a continuous phenotype.To examine the effect of heritability, the relationship between training and validation populations, and the number of categories on the loss in accuracy due to categorizing a continuous trait, the accuracy of GEBV from the analysis of ordinal categorical scores from 1000 or 2250 training animals was expressed relative to the accuracy for the continuous phenotype in Figure 2. This relativity is important because a researcher with a fixed budget may have the choice of investing in either a difficult to measure expensive phenotype or of genotyping a larger training population that is characterized with a cheap and easily measured categorical score. For this reason, to further characterize the loss of information in terms of training population size, when going from a continuous to an ordinal categorical phenotype, the accuracy for the ordinal categorical phenotype from 2250 training animals was expressed relative to the accuracy for the continuous phenotype from 1000 training animals in Figure 3.

**Table 2:** **Correlation between true (*g *) and predicted (*ĝ*) breeding values in the Angus (PV) and multibreed (MV) validation populations**

				$\left(g_{\text{PV}},{ĝ}_{\text{PV}|\text{PT}}\right)$			$r(g_{\text{MV}},{ĝ}_{\text{MV}|\text{PT}})$	
**Type of phenotype**	**Model**	***h***^**2**^	***c***	**n = 1000**	**n = 2250**		**n = 1000**	**n = 2250**
Continuous	Linear	0.10	-	0.12 ± 0.02	0.31 ± 0.02		0.04 ± 0.01	0.23 ± 0.02
		0.25	-	0.28 ± 0.03	0.51 ± 0.02		0.21 ± 0.02	0.41 ± 0.02
		0.50	-	0.55 ± 0.02	0.66 ± 0.02		0.42 ± 0.02	0.53 ± 0.01
Categorical	Threshold	0.10	2	0.04 ± 0.02	0.14 ± 0.02		0.01 ± 0.01	0.09 ± 0.02
			3	0.05 ± 0.01	0.13 ± 0.03		0.03 ± 0.01	0.09 ± 0.02
			4	0.07 ± 0.01	0.14 ± 0.02		0.02 ± 0.01	0.10 ± 0.02
		0.25	2	0.15 ± 0.03	0.29 ± 0.03		0.10 ± 0.02	0.22 ± 0.08
			3	0.15 ± 0.03	0.32 ± 0.03		0.11 ± 0.02	0.24 ± 0.02
			4	0.16 ± 0.03	0.34 ± 0.03		0.10 ± 0.02	0.27 ± 0.02
		0.50	2	0.32 ± 0.02	0.51 ± 0.02		0.26 ± 0.02	0.41 ± 0.02
			3	0.36 ± 0.02	0.53 ± 0.02		0.28 ± 0.02	0.43 ± 0.02
			4	0.38 ± 0.02	0.55 ± 0.02		0.29 ± 0.02	0.44 ± 0.02
Categorical	Linear	0.10	2	0.04 ± 0.01	0.04 ± 0.01		0.01 ± 0.01	0.01 ± 0.01
			3	0.04 ± 0.01	0.04 ± 0.01		0.01 ± 0.01	0.01 ± 0.01
			4	0.03 ± 0.01	0.03 ± 0.01		0.01 ± 0.01	0.01 ± 0.01
		0.25	2	0.09 ± 0.02	0.23 ± 0.03		0.08 ± 0.02	0.18 ± 0.02
			3	0.09 ± 0.02	0.23 ± 0.03		0.06 ± 0.02	0.18 ± 0.03
			4	0.05 ± 0.01	0.24 ± 0.03		0.03 ± 0.01	0.19 ± 0.02
		0.50	2	0.25 ± 0.03	0.46 ± 0.02		0.20 ± 0.02	0.38 ± 0.02
			3	0.26 ± 0.03	0.48 ± 0.02		0.19 ± 0.02	0.38 ± 0.02
			4	0.29 ± 0.03	0.50 ± 0.03		0.20 ± 0.02	0.39 ± 0.02

Results are given for Bayes C *π* linear model analysis of continuous phenotypes and Bayes C *π* linear or threshold model analysis of ordinal categorical phenotypes classified (*c*) as 2, 3 or 4 categories with heritabilities (*h*^2^) of 0.10, 0.25 or 0.50 in the Angus training (PT) population with (n) 1000 or 2250 observations.

### Effect of validation population

There was a substantial difference between actual accuracies of GEBV from PV and MV validation populations. PV validation populations resulted in about 30 to 40% higher accuracy than MV validation populations for ordinal categorical phenotypes (Table 2). The relative accuracy of GEBV for categorical phenotypes in Figure 2 indicated that 5 to 80% of the accuracy from the continuous phenotype could be obtained in the analysis of the ordinal categorical phenotype within PV and MV validation populations. In addition, the relative accuracies of GEBV for categorical phenotypes within PV validation populations were about 1.5 fold higher than those within MV validation populations across heritabilities, training population size, analytical model and number of categories and a 2.25 fold increase in the training population size was not sufficient to provide similar relative accuracies within PV and MV validation populations (Figure 2).Relative accuracies of GEBV for ordinal categorical phenotypes in Figure 3 were equal to or greater than 100% within PV and MV validation populations for heritabilities of 0.25 and 0.50. These findings indicate that a 2.25 fold increase in the size of the training population was sufficient to obtain a similar accuracy of GEBV for continuous and ordinal categorical phenotypes within PV and MV validation populations. However, this increase was not sufficient for a linear model analysis of ordinal categorical data with a heritability of 0.10 within PV and MV validation populations.

These results demonstrate that validation of genomic prediction analyses of ordinal categorical phenotypes is sensitive to the choice of validation population and to pedigree relationships between the animals contributing to validation and training populations as has been shown for continuous traits [6]. Saatchi et al. [6,32] applied genomic prediction to US Angus, Limousin and Simmental beef cattle to evaluate some routinely measured economically important traits. The accuracy of GEBV ranged from 0.22 to 0.69 in Angus, from 0.39 to 0.76 in Limousin and from 0.29 to 0.65 in Simmental cattle, using K-means clustering to minimize relationships between training and validation groups. The accuracy (0.38 to 0.85) of GEBV obtained by random clustering was higher for all traits than the corresponding accuracies obtained by K-means clustering. Villanueva et al. [23] found higher accuracies than this study, equal to about 0.4 or 0.6, in the analysis of binomial phenotypes with a heritability of 0.1 or 0.3 but with higher genetic relationships between the training and validation populations.

The difference between accuracies obtained in PV versus MV validation populations could result from the different extent and patterns of linkage disequilibrium (LD) because there were significant differences in the extent of LD between PT, PV and MV populations (data not shown). Toosi et al. [9] reported that using training and validation populations from the same breed resulted in the highest values of accuracy of GEBV in other cross or admixed populations and that compared to these values, when training and validation were done in different breeds, accuracy dropped by 46%.

### Effects of training population size and heritability

The importance of training population size and heritability on the accuracies of GEBV for continuous phenotypes [2,31] and ordinal categorical phenotypes [23] has been shown in simulation studies. The accuracy of GEBV depends on the genetic variation for the trait analyzed and the number of animals in the training population [33]. An increase in accuracy with training data size was confirmed in real continuous phenotypes. Habier et al. [31] indicated that the accuracy of GEBV improved markedly with training data size for milk yield, fat yield and somatic cell scores from 1000 to 4000 North American Holstein bulls. In a study on the persistence of accuracy of GEBV over generations in layer chickens, Wolc et al. [7] determined that accuracy tended to increase when the number of observations available for the training population increased about five folds from generation 1 to 5.

Table 2 shows that when the training population size and heritability increased, accuracy of GEBV increased significantly for all models of analysis and for all number of categories in the ordinal categorical data. Increasing the size of training population resulted in increased accuracies of GEBV for heritabilities of 0.10 and 0.25 more than for heritabilities of 0.50 within the PV and MV validation populations. The gain in accuracy of GEBV was about 85 to 230% for heritabilities of 0.10, 170 to 210% for heritabilities of 0.25 and 65 to 75% for a heritability of 0.50 across validation populations and analytical models (Table 2). The effect of increasing training population size varied considerably depending on heritabilities, analytical models and the number of categories. The highest gain for a binomial phenotype was observed with heritabilities of 0.10 or 0.50, whereas the highest gain for four category ordinal phenotypes was found with a heritability of 0.25 across validation populations. The same pattern of relationships with training population size and heritability were observed for the accuracy of GEBV for the ordinal categorical phenotype relative to the continuous phenotype (Figure 2). The largest increase in accuracy with training population size was observed for a heritability of 0.1 but the largest increase for relative accuracy was for heritabilities of 0.25 and 0.5 (Figure 2). For heritabilities of 0.25 and 0.50, the accuracy obtained for the categorical trait relative to the continuous trait increased with the number of categories, but this trend was observed neither with a heritability of 0.1, nor for the linear model.With a threshold model, the accuracies of GEBV for ordinal categorical phenotypes given in Figure 3 indicate that a 2.25 fold increase in training population size was sufficient to achieve an accuracy equal to or greater than that obtained for the continuous phenotypes with a training population size of 1000 animals across heritabilities and numbers of categories. However, with a linear model, a greater than 2.25 fold increase in the size of training population would be required to achieve the same accuracy as a continuous trait with 1000 observations for the analysis of an ordinal categorical phenotype with a heritability of 0.10.

### Effects of the analytical model and the number of categories

Accuracies of GEBV in Table 2 and Figures 2 and 3 show that the threshold model had higher accuracies than linear model analyses when analyzing categorical data. Varona et al. [34] compared linear and threshold models in conventional pedigree-based evaluations (EBV) by examining the correlation between predicted and true breeding values using simulated data sets for calving difficulty. The correlations with the threshold model were better than with the linear model for both direct and maternal effects. Ramirez-Valverde et al. [27] compared the accuracy of EBV from threshold animal, threshold sire-maternal grandsire, linear animal and linear sire-maternal grandsire models for calving difficulty in beef cattle and determined that the accuracy of EBV from the threshold model was 10% higher than from the linear model for animal and sire-maternal grandsire models. Casellas et al. [35] analyzed litter size using linear and threshold models and found better goodness-of-fit and predictive ability for EBV from a threshold model than for a linear model.

Table 3 shows the Spearman rank correlations among GEBV (${ĝ}_{\text{Con}},{ĝ}_{T}$ and ${ĝ}_{L}$) in the Angus (PV) and multibreed (MV) validation populations after estimating the substitution effects from the 50k panel without QTL using the Bayes C *π* linear (*Con*) model analysis of continuous phenotypes and Bayes C *π* linear (*L*) or threshold (*T*) model analysis of ordinal categorical phenotypes. The rank correlations between ${ĝ}_{T}$ and ${ĝ}_{L}$ ranged from 0.52 to 0.89 in the PV validation population and from 0.48 to 0.85 in the MV validation population. Categorical phenotypes classified by 2 or 3 scores resulted in higher rank correlations among the alternative analyses than when categorical phenotypes were classified by 4 scores across heritabilities and training population sizes. However, the rank correlations were not affected when heritabilities and training population sizes increased. The rank correlations between ${ĝ}_{\text{Con}}$ and ${ĝ}_{T}$, and between ${ĝ}_{\text{Con}}$ and ${ĝ}_{L}$ indicated that the Bayes C *π* linear (*Con*) model analysis of continuous phenotypes and Bayes C *π* threshold (*T*) model analysis of ordinal categorical phenotypes resulted in GEBV with a similar ranking than the Bayes C *π* linear (*Con*) model analysis of continuous phenotypes and Bayes C *π* linear (*L*) model analysis of ordinal categorical phenotypes across heritabilities, number of categories and training population sizes. Vazquez et al. [36] compared Poisson, logit and linear models for accuracy of EBV for clinical mastitis in Norwegian Red cows. They found that the type of model, linear or nonlinear, had an impact on accuracy and the ranking of sires. Guerra et al. [37] and Marcondes et al. [38] studied linear and threshold models for the analysis of calving rate and calf survival in a multibreed beef cattle population and for the analysis of stayability for Nellore cows and found that the two models resulted in EBV with very similar rankings (rank correlation = 97%).

**Table 3:** **Spearman rank correlation among predicted (**${ĝ}_{\text{Con}},{ĝ}_{T}$** and**${ĝ}_{L}$**) genotypic values**

		$r{\left({ĝ}_{\text{Con}},{ĝ}_{T}\right)}_{\text{PV}}$	$r{\left({ĝ}_{\text{Con}},{ĝ}_{L}\right)}_{\text{PV}}$	$r{\left({ĝ}_{T},{ĝ}_{L}\right)}_{\text{PV}}$	$r{\left({ĝ}_{\text{Con}},{ĝ}_{T}\right)}_{\text{MV}}$	$r{\left({ĝ}_{\text{Con}},{ĝ}_{L}\right)}_{\text{MV}}$	$r{\left({ĝ}_{T},{ĝ}_{L}\right)}_{\text{MV}}$
*h*^2^	*c*	n = 1000	n = 1000	n = 1000	n = 1000	n = 1000	n = 1000
0.10	2	0.26	0.21	0.61	0.29	0.26	0.60
	3	0.27	0.22	0.62	0.31	0.27	0.57
	4	0.32	0.26	0.52	0.34	0.29	0.48
0.25	2	0.34	0.31	0.74	0.33	0.29	0.73
	3	0.37	0.34	0.74	0.37	0.34	0.70
	4	0.40	0.33	0.64	0.40	0.29	0.56
0.50	2	0.54	0.45	0.66	0.50	0.42	0.66
	3	0.59	0.47	0.66	0.54	0.43	0.64
	4	0.61	0.51	0.64	0.55	0.46	0.61
*h*^2^	*c*	n = 2250	n = 2250	n = 2250	n = 2250	n = 2250	n=2250
0.10	2	0.34	0.31	0.89	0.31	0.25	0.83
	3	0.40	0.35	0.88	0.34	0.28	0.82
	4	0.41	0.37	0.82	0.36	0.29	0.74
0.25	2	0.52	0.44	0.86	0.45	0.37	0.85
	3	0.54	0.47	0.84	0.48	0.41	0.82
	4	0.59	0.50	0.80	0.53	0.44	0.80
0.50	2	0.70	0.63	0.85	0.66	0.59	0.84
	3	0.72	0.66	0.87	0.68	0.62	0.84
	4	0.74	0.68	0.87	0.70	0.63	0.84

Results are for Angus (PV) and multibreed (MV) validation populations using Bayes C *π* linear (*Con*) model analysis of continuous phenotypes and Bayes C *π* linear (*L*) or threshold (*T*) model analysis of ordinal categorical phenotypes classified (*c*) as 2, 3 or 4 categories with heritabilities (*h*^2^) of 0.10, 0.25 or 0.50 in the Angus training (PT) population with (n) 1000 or 2250 observations.

### Bias in predictions

The presence of bias in GEBV was evaluated by regressing true (*g*) genotypic values of validation animals on their predicted (*ĝ*) genotypic values (Table 4). These regression coefficients tended to differ from the expected value of 1. Regression coefficients for the MV validation population were lower than those for the PV validation population, regardless of heritability, training population size, model of analysis and the number of categories, when the PT training population was purebred. GEBV were found to be more biased with 1000 observations than with 2250 observations in the training population. Generally, when heritability and training population size increased, bias reduced. The least bias occurred when the phenotype was continuous. The threshold model resulted in greater bias than analysis of a continuous phenotype. Some of the worst bias occurred when the data was categorical but analyzed as if it was continuous using a linear model. Generally the more biased predictions were associated with lower accuracy. Saatchi et al. [6] indicated that traits in US Angus that presented the highest bias i.e. having regressions of deregressed estimated breeding values (DEBV) on DGV different from 1, also exhibited less accuracy, regardless of the number of animals with DEBV.

**Table 4:** **Regression (*b *) of true (*g *) on predicted (*ĝ*) genotypic values**

				$b(g_{\text{PV}},{ĝ}_{\text{PV}|\text{PT}})$			$b(g_{\text{MV}},{ĝ}_{\text{MV}|\text{PT}})$	
**Type of phenotype**	**Model**	***h***^***2***^	***c***	**n = 1000**	**n = 2250**		**n = 1000**	**n = 2250**
Continuous	Linear	0.10	-	0.57 ± 0.11	1.10 ± 0.27		0.33 ± 0.06	0.92 ± 0.25
		0.25	-	1.01 ± 0.27	1.02 ± 0.10		0.73 ± 0.12	0.86 ± 0.09
		0.50	-	0.87 ± 0.07	0.87 ± 0.04		0.73 ± 0.04	0.78 ± 0.05
Categorical	Threshold	0.10	2	0.28 ± 0.13	1.11 ± 0.43		0.05 ± 0.05	0.90 ± 0.45
			3	0.08 ± 0.09	0.45 ± 0.16		0.02 ± 0.04	0.34 ± 0.09
			4	0.52 ± 0.16	0.55 ± 0.21		0.23 ± 0.12	0.35 ± 0.10
		0.25	2	0.81 ± 0.23	1.00 ± 0.24		0.65 ± 0.16	0.87 ± 0.14
			3	0.61 ± 0.29	1.34 ± 0.21		0.63 ± 0.16	1.03 ± 0.17
			4	1.12 ± 0.29	1.21 ± 0.18		0.66 ± 0.12	1.16 ± 0.30
		0.50	2	0.44 ± 0.11	0.74 ± 0.14		0.43 ± 0.06	0.69 ± 0.07
			3	0.69 ± 0.12	0.87 ± 0.11		0.50 ± 0.07	0.77 ± 0.06
			4	0.56 ± 0.10	1.04 ± 0.06		0.49 ± 0.07	0.82 ± 0.05
Categorical	Linear	0.10	2	0.41 ± 0.18	0.62 ± 0.40		0.10 ± 0.23	0.74 ± 0.40
			3	0.01 ± 0.20	0.86 ± 0.38		0.08 ± 0.08	1.83 ± 0.71
			4	0.18 ± 0.23	0.75 ± 0.27		0.04 ± 0.10	0.50 ± 0.10
		0.25	2	0.63 ± 0.46	2.27 ± 0.51		0.31 ± 0.22	2.07 ± 0.31
			3	0.61 ± 0.38	2.27 ± 0.45		0.15 ± 0.24	1.87 ± 0.32
			4	0.59 ± 0.30	1.91 ± 0.34		-1.24 ± 1.21	1.59 ± 0.40
		0.50	2	4.41 ± 0.64	4.67 ± 0.36		3.94 ± 0.60	3.94 ± 0.29
			3	3.77 ± 0.61	4.11 ± 0.35		3.01 ± 0.48	3.38 ± 0.27
			4	2.85 ± 0.58	3.04 ± 0.21		1.91 ± 0.29	2.31 ± 0.15

Results are for Angus (PV) and multibreed (MV) validation populations using Bayes C *π* linear model analysis of continuous phenotype and Bayes C *π* linear or threshold model analysis of ordinal categorical phenotype classified (*c*) as 2, 3 or 4 categories with heritabilities (*h*^2^) of 0.10, 0.25 or 0.50 in the Angus training (PT) population with (n) 1000 or 2250 observations.

Table 5 shows the estimates of heritabilities on the underlying scale across heritabilities, training population size, and number of categories. Bayes C *π* linear model analysis of continuous phenotypes resulted in downward bias of heritability estimates for training population sizes with 1000 and 2250 observations, except for a heritability of 0.10 and a training population size with 1000 observations. Increasing training population size from 1000 to 2250 did not help improve the estimates of heritabilities. Bayes C *π* threshold model analysis of ordinal categorical phenotypes resulted in upward or downward bias of heritability estimates on the underlying scale for training population sizes of 1000 or 2250. The increase in training population size resulted in similar estimates of heritabilities on the underlying scale from Bayes C *π* linear model analysis of continuous phenotypes and Bayes C *π* threshold model analysis of ordinal categorical phenotypes. However, the estimates of heritabilities on the underlying scale from Bayes C *π* linear model analysis of ordinal categorical phenotypes were found to be significantly downward biased for training population sizes with 1000 and 2250 observations.

**Table 5:** **Estimates of heritability (*h***^**2**^**)**

**Type of phenotype**	**Model**	***h***^***2***^	***c***	**n = 1000**	**n = 2250**
Continuous	Linear	0.10	-	0.16 ± 0.019	0.04 ± 0.004
		0.25	-	0.14 ± 0.018	0.16 ± 0.012
		0.50	-	0.39 ± 0.016	0.39 ± 0.011
Categorical	Threshold	0.10	2	0.56 ± 0.045	0.03 ± 0.003
			3	0.44 ± 0.042	0.03 ± 0.004
			4	0.37 ± 0.043	0.03 ± 0.004
		0.25	2	0.28 ± 0.039	0.10 ± 0.014
			3	0.27 ± 0.040	0.11 ± 0.013
			4	0.25 ± 0.037	0.11 ± 0.013
		0.50	2	0.62 ± 0.047	0.41 ± 0.018
			3	0.60 ± 0.045	0.39 ± 0.017
			4	0.62 ± 0.045	0.40 ± 0.017
Categorical	Linear	0.10	2	0.27 ± 0.037	0.01 ± 0.001
			3	0.26 ± 0.032	0.01 ± 0.001
			4	0.26 ± 0.031	0.01 ± 0.002
		0.25	2	0.09 ± 0.032	0.02 ± 0.004
			3	0.10 ± 0.030	0.03 ± 0.005
			4	0.11 ± 0.028	0.03 ± 0.006
		0.50	2	0.08 ± 0.009	0.10 ± 0.009
			3	0.10 ± 0.010	0.13 ± 0.010
			4	0.13 ± 0.011	0.15 ± 0.011

Estimates were obtained using Bayes C *π* linear model analysis of continuous phenotypes and Bayes C *π* linear and threshold model analyses of ordinal categorical phenotypes classified (*c*) as 2, 3 or 4 categories with heritabilities (*h*^2^) of 0.10, 0.25 or 0.50 in the Angus training (PT) population with (n) 1000 or 2250 observations.

### Other factors that influence the results from categorical analyses

In some practical settings, linear model analyses perform as well as threshold model analyses as discussed above. This is expected because as the number of categories increases, the distribution of the data tends towards a normal distribution. The worst scenario is for binomial data and although not significant, the trends for accuracy of predictions tend to be poorer for two compared to three or four categories, as in Figure 2. The performance of analyses of binomial data treated as continuous also eroded as the observed frequency departed from 0.5. In the simulations presented here, the distributions of ordered categorical scores were chosen to reflect those commonly observed, namely a majority of observations in one extreme category, and successively fewer scores in each successive category. In data in which the distribution of scores is spread more evenly, the loss in accuracy from using categorical scores rather than measuring a continuous variable will be smaller than that observed here, which indicates that it will not be necessary to increase training population size by as much as 2.25 fold to achieve the accuracy for continuous phenotypes with a training population size of 1000 animals.

The distribution of outcomes across categories can also differ between fixed class variables such as herd-year-season. The simulation reported here used only one fixed class effect with three similarly represented levels. It is difficult to simulate data to represent all situations that might be encountered with field data, but we believe the parameters we have chosen will provide indicative values for relative information content of continuous data compared to that measured with categorical scores.

## Conclusions

Genomic prediction of ordinal categorical phenotypes was carried out using Bayes C *π* threshold and linear models. Results indicated that there was a clear loss in accuracy of GEBV from the analysis of ordinal categorical phenotypes compared to that of continuous phenotypes. This loss was found to depend on training population size, heritability, model of analysis and number of categories. The accuracies of GEBV for ordinal categorical phenotypes analyzed by the threshold model were higher than those with a linear model applied to the scores, and the advantage of a threshold model was greatest when training populations were small. Accuracy of GEBV in a purebred validation population was greater than in a multibreed validation population; however, this difference became smaller when training population size increased. A 2.25 fold increase in training population size for ordinal categorical phenotypes analyzed using a threshold model was sufficient to achieve an accuracy equal to or greater than that for continuous phenotypes with a training population size of 1000 animals.

## Competing interests

The authors declare that they have no competing interests.

## Authors’ contributions

KK developed the marker-based threshold model. KK, RLF and DJG implemented the threshold extension in GenSel software and KK carried out the statistical analysis. KK, RLF and DJG drafted the manuscript. All authors read and approved the final manuscript.
